# Ultralow-Volume Treatments for Mosquito-Borne Diseases

**DOI:** 10.1001/jamahealthforum.2026.1553

**Published:** 2026-07-02

**Authors:** Randall J. Nett, Andrea M. McCollum, Casey Parker-Crockett

**Affiliations:** Division of Vector-Borne Diseases, US Centers for Disease Control and Prevention, Fort Collins, Colorado.

Mosquito management products composed of organophosphates and pyrethroids may target and kill adult, flying mosquitoes (these products are also known as adulticides). The use of aerial- and ground-based ultralow-volume (ULV) treatments of organophosphates and pyrethroids is an essential public health tool in the US to prevent and control mosquito-borne disease outbreaks and protect people. A 2020 survey found 79% of responding mosquito control programs conduct routine vector control, which includes methods such as ULV treatments.^1^ This tool is especially vital considering the public’s low adherence to personal protective behaviors (eg, use of insect repellent) and paucity of approved vaccines for endemic mosquito-borne diseases. Public health leaders should consider the use of ULV treatments to protect communities from mosquito-borne diseases because available evidence demonstrates these interventions are both effective and safe.

Mosquito-borne diseases cause substantial morbidity and mortality each year in the US, and the frequency and size of outbreaks are increasing. Arthropod-borne viruses (ie, arboviruses) can cause severe illness, including neurological disease and death. West Nile virus (WNV) is the most common domestic arboviral disease. During 2014 to 2023, nearly 2000 reported WNV disease cases, including approximately 1300 severe neuroinvasive disease cases and 130 deaths, were reported yearly in the US.^2^ However, reported disease cases are an underestimate of the true burden because many patients do not seek care or are not tested for WNV infection. Examples of other notable domestic mosquito-borne arboviruses in the continental US include Eastern equine encephalitis virus, Jamestown Canyon virus, and La Crosse virus. Other mosquito-borne pathogens represent a risk for importation and spread in the US. Examples include local transmission of pathogens that cause dengue and malaria in several states, Japanese encephalitis in California, and chikungunya and Zika in US territories and states. Local mosquito control responses are vital to control further transmission in local mosquito populations and prevent nonendemic pathogens from emerging in the US.

Aerial- and ground-based ULV treatments are the primary methods for applying mosquito management products against adult mosquitoes in the US. Average droplet sizes range from 5 to 60 μm for ULV treatments and are delivered when mosquitoes are most active and optimized to maximize contact with flying mosquitoes while minimizing nontarget exposure. Given their small size, droplets function as a nonresidual application, meaning they dissipate from the environment rapidly depending on environmental conditions.

ULV treatments are an integral part of integrated mosquito management^3^ and typically used in response to local surveillance signals indicating an increased risk of human disease transmission. ULV treatments allow for rapid suppression of infected adult mosquitoes and interruption of transmission cycles. Additionally, ULV treatments are critical in responding to natural disasters (eg, hurricanes), which can create conditions conducive to explosive mosquito populations.

Evidence supports the effectiveness and safety of aerial and ground ULV treatments. During the 2005 WNV outbreak in California, the likelihood of WNV cases was 6 times higher in comparable untreated areas compared with ULV-treated areas (*P* < .001).^4^ Similarly, during the 2016 Zika outbreak in Florida, the application of mosquito management products targeting larvae and adults led to rapid decreases in *Aedes aegypti* abundance and was associated with interruption of local Zika virus transmission.^5^

Three recent reviews failed to demonstrate that the community ULV application of organophosphates and pyrethroids for mosquito control adversely impacted human health.^6–8^ One study noted an ecological association between pyrethroid use and childhood developmental disorders but could not demonstrate causality because of limitations in study design. The studies were not able to address causality and were limited by inclusion of underpowered study designs that prevented definitive study conclusions, recall biases, availability of noninvasive biomonitoring to allow for quantification of insecticide exposure, reliance on data from emergency or inpatient settings and bias toward more severe outcomes, lack of data for chronic conditions, and potential confounding by personal or agricultural exposure to organophosphates and pyrethroids. At present, the large public health burden caused by mosquito-borne diseases might outweigh any potential health burden caused by adverse health outcomes following community application of products targeting adult mosquitoes based on available and limited evidence. However, additional data are needed to further evaluate the public health impact and safety of mosquito management products targeting adult mosquitoes, which can be accomplished by planning evaluations a priori rather than only post hoc.

Public opinions regarding ULV treatments vary across communities and can impact public health decision-making. For example, strong public sentiment against use of ULV treatments in a community can result in public health authorities choosing to rely on less effective methods. During the 2012 WNV epidemic in north-central Texas, 2 of 4 counties chose not to use aerial ULV treatments because of public concerns.^9^ During the 2016 Zika virus epidemic, application of an organophosphate was delayed by 24 hours because of community protests.^9^ Orange County, California, residents voted down a referendum that would have allowed the mosquito control agency’s manager to conduct aerial ULV treatments to prevent an outbreak.^9^ Support for using mosquito management products does exist in some communities. A survey conducted in Texas suggested that respondents were willing to pay $53.15 per year to expand mosquito control activities in their area.^10^ In a survey of US households during the Zika virus epidemic, those who had confidence the government could address the Zika outbreak were nearly twice as likely to support outdoor application of mosquito management products, reinforcing the need for public health authorities to establish trust in the community as a requisite for using the most effective mosquito control methods.^9,10^

To establish trust in advance of an urgent response, public health programs should consider working closely with community members, local government authorities, private entities, and commercial stakeholders to understand the factors impacting support for or opposition to using ULV treatments in their community. Transparent messaging is needed regarding known risks and benefits of larger-scale ULV treatments compared with those of targeted ULV treatments only, no use of ULV treatments, disease incidence, and the spectrum of clinical and public health outcomes should disease transmission continue. Public health programs need to be prepared to provide and lead evidence-based discussions on these complex topics with stakeholders, including addressing uncertainties. These discussions and frameworks cannot follow a one-size-fits-all approach for communities. Instead, frameworks and decisions should be the best fit for a community or region, reflecting local concerns and agreement for action.

To increase support for use of ULV treatments when needed across jurisdictions, such as when the risk for mosquito-borne disease outbreaks is high, public health programs can implement a communications and community engagement strategy that is both available and tailorable to local health authorities and individual communities. Finally, continued efforts to develop innovative mosquito control technologies are needed to improve the effectiveness and safety of this critical public health tool.
